# Natural Products for the Management and Prevention of Breast Cancer

**DOI:** 10.1155/2018/8324696

**Published:** 2018-02-26

**Authors:** Sarmistha Mitra, Raju Dash

**Affiliations:** ^1^Department of Pharmacy, University of Chittagong, Chittagong 4331, Bangladesh; ^2^Department of Biochemistry and Biotechnology, University of Science and Technology Chittagong, Chittagong 4202, Bangladesh

## Abstract

Among all types of cancer, breast cancer is one of the most challenging diseases, which is responsible for a large number of cancer related deaths. Hormonal therapy, surgery, chemotherapy, and radiotherapy have been used as treatment of breast cancer, for a very long time. Due to severe side effects and multidrug resistance, these treatment approaches become increasingly ineffective. However, adoption of complementary treatment approach can be a big solution for this situation, as it is evident that compounds derived from natural source have a great deal of anticancer activity. Natural compounds can fight against aggressiveness of breast cancer, inhibit cancerous cell proliferation, and modulate cancer related pathways. A large number of research works are now focusing on the natural and dietary compounds and trying to find out new and more effective treatment strategies for the breast cancer patients. In this review, we discussed some significant natural chemical compounds with their mechanisms of actions, which can be very effective against the breast cancer and can be more potent by their proper modifications and further clinical research. Future research focusing on the natural anti-breast-cancer agents can open a new horizon in breast cancer treatment, which will play a great role in enhancing the survival rate of breast cancer patients.

## 1. Introduction

Breast cancer is an important public health problem worldwide [1], which is the second most common cancer diagnosed and is also a prime reason of death in women globally [2]. It has become a fatal disease, and risk factors associated with breast cancer seem to be expanding day by day [3]. A number of exogenous and endogenous factors can stimulate the pathology of breast cancer and can worsen the situation [4]. Some additional factors like side effects of conventional treatment such as chemotherapy [5] and radiotherapy [6] are increasing the burden more and making it more challenging to treat the patients of breast cancer. One of the most threatening problems with conventional treatment is multidrug resistance (MDR) [7, 8]. Due to this reason, the survival rate of breast cancer patient is very unsatisfactory. In order to face this deadly situation, many research works are now designed dedicatedly to find out the alternative treatment system of breast cancer which can be used as therapeutics, as an adjuvant treatment besides other treatments, or as chemopreventive agents [9–12]. The etiology of breast cancer and proliferation of cancerous cells is generally mediated by a number of mechanisms or pathways [13–16]. Again there are a number of biomarkers used for diagnostic process [17]. Any of these mechanisms can be targeted by a compound derived from natural source and can be established as a treatment approach [18]. Till now, more than 50% of drugs have been designed from natural compounds and, among them, 75% of anticancer drugs were designed and developed from the plant derived natural ingredients [19].

Natural products derived from a variety of sources may have the tendency to stimulate many physiological pathways which can be beneficial for ailment of stubborn diseases [20] like cancer [21]. For many years cancer remains a very complex disease to treat effectively; the application of different strategies to treat cancer becomes mandatory. To solve this complex puzzle, plant derived compounds are explored. A great number of researches have been carried out to design cancer therapeutics from natural compounds especially from phytochemicals [22, 23]. Recent studies suggested that natural compounds from dietary sources can target a number of pathways related to breast cancer, which can give a positive feedback against malignancies and can also play a key role in preventing breast cancer [24]. In this review, some critical and significant mechanisms related to breast cancer are highlighted, and, on the basis of the key components of the mechanisms, some phytochemicals are discussed and their mechanisms and potentiality to fight with breast cancer are featured. The mechanisms of breast cancer development like epigenetic modifications, aromatase activity, arachidonic acid pathway and cell apoptosis pathway, and natural compounds acting against these mechanisms are discussed. These phytochemicals can mitigate the harmful adverse effects of conventional treatment [25] and also help a patient to fight with mental distress and anxiety. From many studies, it has been established that natural compounds from plants as well as from dietary sources can be very helpful to treat breast cancer, and also many compounds are suggested to work with great efficacy. Therefore, this work is trying to accumulate the most effective natural compounds which can control one or more than one of these pathways and become a successful potential treatment approach.

## 2. Different Subtypes of Breast Cancer

**Figure 1:**
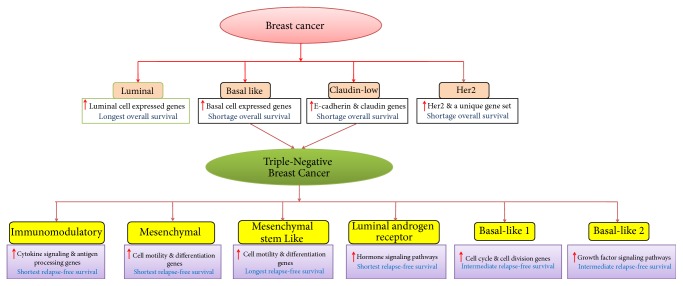
Schematic representation of molecular subtypes of breast cancer (Perou et al. [26–28] denoted by pink color) and triple-negative breast cancer types (Lehmann et al. [29] denoted by yellow colour). Red arrow mark indicates the higher expression of cellular gene in each subtype. The subtypes are characterized by overall survival (for breast cancer subtypes) and relapse-free survival (for TNBC subtypes).

Basically, ductal hyperproliferation is the initial stage of breast tumor development, where different carcinogenic factors play major roles to turn this stage into benign tumors or even metastatic carcinomas [30]. According to the genome profiling of breast tumors, several molecular forms of breast cancer were first classified by Perou et al. [26, 27] concluding the four primary subgroups of breast tumor (shown in Figure 1). Gene overexpression and overall survival are the two main factors to characterize these subtypes, as overexpressions of genes are normally associated with luminal and basal-like subtypes and also correlated with the longest and shortest survival, respectively, while HER2 subtype is associated with the overexpression of EGFR and unique set of genes is also correlated with the short survival. It should be noted that the responsible drivers for the poor survival of basal-like tumors still remain unclear. Later, Prat et al. [28] reported another subtype claudin low tumor that is associated with high expression of epithelial-to-mesenchymal transition markers, immune response genes, and cancer stem cell-like features. However, this subtype shows low to absent expression of luminal differentiation markers, which is correlated with decreased survival [31]. Interestingly, both of these subtypes have the high molecular heterogeneity and significantly cover the common characteristics of triple-negative breast cancers (TNBCs). The TNBCs are highly aggressive in nature and generally characterized as the lacking expressions of three biomarkers, that is, ER, PR, and HER2 proteins. The development of targeted therapy in TNBC is very challenging, though Lehmann et al. [29] reported six different TNBC subtypes. Each subtype of breast tumor would respond differently to treatment, which made breast cancer extremely intractable [32]. As described in Figure 1, each TNBC subtype represents different gene expressions and survivals, which make it more challenging to define accurate selection of chemotherapeutics or drugs that are both effective and safe to breast cancer patient.

## 3. Conventional Treatment and Its Drawbacks

Presently applicable treatment strategies for patients of breast cancer include surgery, radiotherapy with an adjuvant chemotherapy, and hormone therapy which can provide positive feedback [33]. Two early stages of breast cancer, stages I and II, are usually treated with breast-conserving surgery and radiation therapy. Radiation therapy following breast-conserving surgery reduces the risk of death and recurrence [34]. However, it has been reported that there can be an incidence of brachial plexopathy, rib fracture, tissue necrosis, pericarditis, and second non-breast infield malignancies occurring in patients with early stage breast cancer treated with surgery and radiation therapy [35]. In case of TNBC treatment, the only successful and systemic therapy is “chemotherapy.”

Multidrug resistant (MDR) tumors are another threat towards cancer therapy and are a very serious cause of cancer related deaths in patient. Therefore, understanding the molecular basis of MDR and developing medicines as well as treatment regimens to prevent drug resistance is an important priority [36]. A number of complex mechanisms can be directly related to the occurrence of drug resistance, including modification of drug efflux membrane transporters, namely, P-glycoprotein, MRP 1, BCRP [37, 38], and alterations in beta-tubulin [38]; multidrug resistance protein (MRP) family consists of 9 members (MRP1–9) [39]. Many drugs which were previously very successful such as anthracyclines (doxorubicin, daunorubicin, epirubicin, and mitoxantrone), taxanes (paclitaxel, docetaxel), and capecitabine can be resistant to a patient [40], and it is suggested by Fumoleau et al. to administer monotherapy in breast cancer treatment. Again there is a fact that cancer stem cells are immune to any therapies and they have the ability to maintain “stemness.” It is their unique ability to continue to populate tumor mass again and again with an increasing supply of new cancer cells [41].

Furthermore, major side effects of chemotherapy may lead to the reduction of white blood cells and red blood cells thus increasing the possibility of infection and anemia, respectively, with reduced oxygen carrying capacity of the cells. Another significant side effect is hair loss resulting from conventional treatment. Fatigue, sore throat, nausea, ulcers, loss of appetite, change in taste, constipation, diarrhea, change in skin color, and various hormonal changes are other side effects which are also observed during these treatments [42].

On the basis of all these understandings, finding out an alternative approach of breast cancer management is an urgent need that can avoid and minimize the risks of unusual side effects of conventional treatment approaches.

## 4. Herbal Approach in Treatment of Breast Cancer

**Figure 2:**
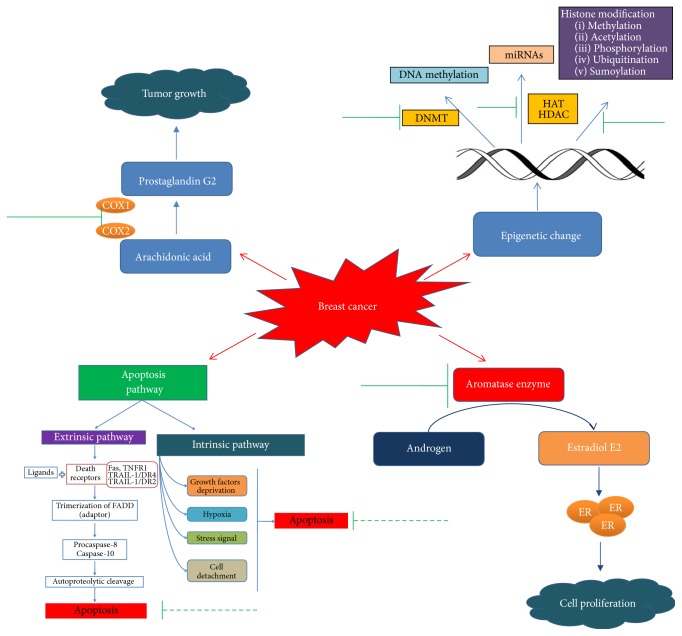
Molecular targets of natural compounds in breast cancer pathway. Here green solid line indicates the action of natural compounds as inhibitor, while green dotted line represents the upregulation induced by natural compounds.

Natural compounds or phytochemicals provide positive health benefits by acting directly on specific molecular targets such as genes, or by indirectly stabilizing conjugates that affect metabolic pathways [43]. Liao et al. in 2013 reported that herbal compounds can be effectively used as adjuvants with conventional chemotherapy with a view to reducing side effects like fatigue, nausea, mucositis, and anemia arising from chemotherapy or other treatments [44]. From many studies, it is evidenced that many of the natural compounds have anticancer activity and they have the property to function as a treatment approach via many mechanisms (Figure 2) [45–48]. In the early genesis of cancer and progression, epigenetic dysregulation is mostly observed besides genetic changes. Epigenetic modification involves changing of cellular phenotype without involvement of modification of underlying DNA sequence. The mechanisms comprise the DNA methylation, mainly at cytosines (creating the 5-methylcytosine) when positioned next to a guanine (CpG dinucleotides), methylation, acetylation, phosphorylation, ubiquitination, and sumoylation of histones and noncoding RNAs or miRNAs that affect the expression of mammalian genome [49]. In breast cancer, abnormal histone modifications like acetylation and methylation of histone along with DNA hypermethylation are associated with epigenetic silencing of tumor suppressor genes and genomic instability [50, 51]. In this context, the readers are suggested to refer to some recent reviews describing the functional roles of epigenetics and the possible epigenetic targets which altered expressions and are commonly associated with the breast cancer development and progressions [52, 53]. Accumulating evidences showed that natural phytochemicals including the secondary metabolites found in the dietary foods have the ability to modulate the epigenetic events and reverse the epigenetic changes before causing cancer progression [54, 55]. By targeting specific key transcription factors, kinases, and growth factor receptor mediated pathways, phytochemicals are reported to exert their actions in epigenetic alterations through cell cycle arrest, initiating apoptosis and reactivation of tumor suppressing genes [56, 57]. In breast cancer chemoprevention, the article reported by Chlebowski [58] showed the effective chemopreventive actions of tamoxifen and some aromatase inhibitors including exemestane and anastrozole in reduction of breast cancer on clinical trial studies. However, these drugs showed favorable side effects, including tamoxifen, that increase the greater risk of endometrial cancer. Compared to the synthetic drugs, natural compounds are very much promising in breast cancer chemoprevention, as they showed less side effects and minimal toxicity in both* in vitro* and* in vivo* experiments [59]. Aromatase, a membrane bound protein and a member of the cytochrome P450 enzyme family, catalyzes the conversion of androstenedione to estrone (E1) and of testosterone to estradiol (E2) and plays a very significant role in biosynthesis of estrogen [60]. A number of recent studies showed that phytochemicals have the similar chemical structure to estrogen and have the ability to alter aromatase expression by directly inhibiting the aromatase activity [61]. Furthermore, evidences also reported that natural products also show chemopreventive actions by targeting arachidonic acid (AA) pathway, including metabolic enzymes cyclooxygenases (COXs), phospholipase A2s (PLA2s), and lipoxygenases (LOXs) [62]. Many studies reported that AA pathway plays a key role in the inflammation and also in tumorigenesis [63, 64]. A highly positive association was reported between the high level of COX-2 and poor prognosis, invasiveness, and density of breast cancer cells [65]. Ranger et al. found a positive correlation between the expression levels of COX-2 and distant metastases in breast cancer [66]. By considering this, it was experimented and later found that knocking down COX-2 can eventually reduce the metastatic behavior of breast cancer cells in mice [67]. To get more insight in these two pathways, reader is suggested to read the recent reviews on arachidonic acid metabolite by Borin et al. [68] and aromatase inhibitors by Chumsri et al. [69]. A number of phytochemicals such as curcumin, ginsetin, lycopene, and apigenin have been directly reported to inhibit biosynthesis of metabolic products such as prostaglandins and leukotrienes and, therefore, have been considered as the potent therapeutic agents in breast cancer chemoprevention.

Findings also suggested that phytochemicals have significant effects to stimulate apoptosis in malignant and premalignant cells both* in vitro* and* in vivo* [70]. Apoptosis refers to the programmed cell death that plays crucial roles for embryonic development and tissue homeostasis, and its dysregulation may cause tumor formation or even development of cancer cell drug resistance [71]. Therefore, evasion of apoptosis is considered as one of the most significant approaches to find out and design cancer treatment strategy [72]. Two main pathways related to the activation of apoptosis are extrinsic and intrinsic pathway. The extrinsic pathway starts when the ligand binds with the death receptor and ultimately triggers the trimerization and causes the recruitment of the adaptor protein Fas-associated death domain (FADD) and procaspase-8 and/or caspase-10 to form a death-inducing signaling complexes (DISC) in the intracellular death domain [73, 74]. The death receptors are including Fas (CD95/APO-1), TNF-receptor 1 (TNF-R1/p55/CD120a), TNF-related apoptosis-inducing ligand receptor 1 TRAIL-R1/death receptor 4 (DR4), and receptor 2 (TRAIL-R2/DR5/APO-2/KILLER) [75, 76]. In the DISC, caspase-8 or caspase-10 oligomerization activated DISC through autoproteolytic cleavage that leads to triggering of the enzymatic activity of downstream effector caspases, such as caspase-3 and caspase-7 [77]. In contrast, the intrinsic mitochondrial pathway is activated by physical or chemical stimulations, such as hypoxia, growth factor deprivation, cell detachment, or stress signals, where the proapoptotic Bcl-2 family members play major roles in the initiation of this pathway. [78]. Caspase-9 is also associated with the intrinsic pathway of apoptosis. Once the pathway is activated, caspase-9 cleaves and activates the downstream effector caspase-3 and caspase-7, which then cleaves the key regulatory and structural proteins to execute cell death. Most extensive studies reported that many tumor promoter proteins inhibit apoptosis, by developing chemoresistance in cancer cell. Thereby targeting proteins that manipulated the apoptotic programs is considered as prominent anticancer drug targets, in which activation of apoptosis in cancer cells is the primary concern [79, 80]. Currently, many studies are focusing on the natural compounds that have been approved for the clinical use in the treatment of cancer to find out their ability to inhibit the growth of cancer cells by inducing apoptosis through one or more than one mechanism [81, 82].

## 5. Natural Compounds as Therapeutics

### 5.1. 3,3′-Diindolylmethane

3,3′-Diindolylmethane (DIM) is a natural compound that is abundant in cruciferous vegetables such as broccoli, cauliflower, and cabbage and it is a major acid condensation product of indole-3-carbinol (I3C) [170]. In acidic condition, I3C is converted to DIM in the stomach [171]. Experimental evidences suggested that DIM inhibited COX-2 expression induced by aryl hydrocarbon receptor in human breast cancer cell [83]. Fan et al. [172] demonstrated that DIM stimulated the phosphorylation of Brca1 during the oxidative stress and played protective roles. Further studies showed that DIM inhibits the expression of angiogenesis expressing genes including surviving [84] and hypoxia-inducible factor-1 [85]. In combination therapy with herceptin, DIM reduced the expression of FoxM1 in HER-2/Neu-expressing breast cancer cells through downregulating Akt and NF-kB p65 [87]. Similar result is also observed by Ahmad group in case of Taxotere, in which DIM also targeted FoxM1 [88]. Consequently, in a randomized, placebo-controlled trial studies, DIM significantly increased the chemosensitivity of tamoxifen and showed favorable effect on estrogen metabolism [89]. Similarly, Wang et al. showed that DIM sensitized *γ*-radiation and induced apoptosis through cell cycle arresting at G2/M phase and also increased intracellular ROS generation [173]. DIM also upregulates the expression of* CYP19* in MDA-MB-231 cells and decreases aromatase expression in MCF-7 cells thereby acting as an aromatase inhibitor [86]. Significant tumor inhibition by DIM was also observed in rodent model [174, 175]. Despite significant efficacy of DIM on breast cancer treatment, Marques et al. [176] recently showed that DIM induced cellular proliferation at concentration of 10 *μ*M by activating estrogen receptor *α* signaling pathways, in the absence of estradiol, indicating adverse risk of taking DIM as dietary supplement. However, further research should be needed to understand the unexpected adverse effects of DIM supplement for the treatment or prevention of breast cancer.

### 5.2. Biochanin A

Biochanin A is an isoflavone extracted from red clover (*Trifolium pratense*) which is known to have anticancer activity [177]. It was reported by Wang et al. [90] that, by assaying MCF-7 cells stably transfected with CYP19 gene, biochanin A blocked the activity of aromatase enzyme and stopped cell growth attributing to the enzyme activity. Furthermore, in SK-BR3 cells (ER-negative breast cancer cells), biochanin A was found to inhibit aromatase enzyme activity and reduce mRNA expression. Genistein is a metabolite of biochanin A, which was also found to suppress promoter I.3/II activation and function as an AI. It was reported that biochanin A is tolerated better than genistein and causes positive expression of tumor suppressor genes in HMEC, MCF 12A, and MCF7 (ER-positive breast cancer cell line) cell lines [91, 178]. Similar finding was also observed by Young et al., in which biochanin A showed better induction on tumor suppressor gene expression compared to genistein [179]. Bhushan et al. reported that biochanin A inhibited cell viability, signaling pathways, and invasive enzyme expression and activity in SK-BR-3 cancer cells [91]. Another study done by Moon et al. in xenograft mouse model showed that biochanin A was effective to decrease the growth of estrogen-dependent MCF-7 tumors at doses of 5 or 15 mg/kg per day [92]. Nevertheless, the effect of biochanin A in the other pathways of breast cancer progression still needs to be clarified and the further studies in clinical trial settings are also required to understand the bioavailability and the therapeutic regimen of biochanin A and its metabolic profile in different breast cancer patient's types. As most of the experimental studies are conducted in ER-positive cell line, particular role of biochanin A on ER-negative or triple-negative breast cancer cells should be focused on more in future researches.

### 5.3. Curcumin

Curcumin, the active ingredient of turmeric, is known as a polyphenolic compound and has a very widespread medicinal activity including anti-breast-cancer activity. Curcumin can exert its activity by mediating a number of pathways. Curcumin is reported to induce breast cancer apoptosis by regulating the expression of apoptosis related genes and proteins [180]. Recent works suggest that curcumin can induce apoptosis in breast cancer cell by enhancing the level of p53 which in turn induces Bax expression, leading to an elevated Bax/Bcl-2 ratio. This cascade of events leads to programmed death of breast cancer cells [181].

It was reported that curcumin can downregulate NF-*κ*B expression which is a signaling molecule playing important part in cell proliferation. By reducing expression of NF-*κ*B curcumin can exert antiproliferation effect on MDA-MB-231 and BT-483 cells [93]. Another study suggested that curcumin can decrease the protein expression of urokinase-type plasminogen activator via NF-*κ*B activation which can ultimately stop the adhesion and invasive nature of MCF-7 cells and can suppress metastatic progression of breast cancer [182]. A number of works studied and evaluated the impact of curcumin on NF-*κ*B signaling which can be explored for further study [183, 184].

Curcumin can also show its activity on cancer stem cells which can be a potential strategy of breast cancer treatment. Kakarala et al. [94] reported that curcumin inhibited Wnt signaling in MCF7 cells which is dysregulated in breast cancer [185, 186]; this pathway plays an important role in breast stem cell self-renewal. By inhibiting this pathway, curcumin is proved to be a very promising agent for anti-breast-cancer treatment.

Again it shows epigenetic activity which can be a treatment field. Curcumin is a potential modulator of histones and it regulates enzymatic function of HATs and HDACs. It is evident that curcumin blocks the expression of class I HDACs [95]. Additionally it upregulates the expression of some miRNAs involved in carcinogenesis to reduce the expression of Bcl-2 [96].

Curcumin can also enhance the activity of chemotherapeutic agents. Curcumin inactivates NF-*κ*B expression and this can enhance the efficacy of paclitaxel. This combined effect minimizes breast cancer growth in MDAMB-231 (ER−/PR−) cells. This combined therapy of paclitaxel and curcumin decreases size of tumor and tumor cell proliferation with increased rate of apoptosis and downregulates MMP-9 expression [97]. Curcumin also shows synergistic effect with lots of other compounds which are discussed in other review works [43]. Curcumin can also act against the difficulty of multidrug resistance [187]. Limtrakul et al. [98] found the inhibitory activity of tetrahydrocurcumin on ATP-binding cassette (ABC) drug transporters, including P-glycoprotein (ABCB1/P-gp), multidrug resistance protein 1 (ABCC1), and mitoxantrone resistance protein (ABCG2/MXR) which proved that curcumin is a potential chemosensitizer and acts against drug resistance.

It can be suggested that curcumin is one of the most significant compound to face the challenges of breast cancer treatment. However, the problem of curcumin is its poor bioavailability, which is a great obstacle in developing a therapeutic [188]. Hence, future works should focus on designing and developing more potent analogues of curcumin to overcome the drawbacks of poor bioavailability.

### 5.4. Epigallocatechin Gallate

Epigallocatechin gallate (EGCG) is one of the most phenolic catechins present in green tea and is widely known for its health-related benefits [189]. It is found that EGCG has epigenetic effects in carcinoma cell line either by demethylation or suppressed methylation of promoters of tumor suppressor genes [100, 101]. Li et al. [190] demonstrated in a recent work that EGCG in combination with class I HDAC inhibitor, trichostatin A (TSA), could synergistically reactivate ER*α* expression in ER*α* negative MDA-MB-231 breast cancer cells by modulating histone methylation and acetylation pattern at the gene promoter. It is reported by Deb et al. in 2015 [102] that treatment of breast cancer cells with EGCG could induce the expression of epigenetically repressed TIMP-3 gene. The TIMP-3 gene was mediated through modulation of epigenetic mechanisms which includes EZH2 and class I HDACs independent of the promoter DNA methylation. After treating with EGCG, the protein levels of class I HDACs and EZH2 were reported to reduce to a great level.

Furthermore, Goodin et al. [191] suggested that EGCG inhibited the proliferation of estrogen-sensitive MCF-7 breast cancer cell line and also binds to both ER*α* and ER*β* [192]. Induction of apoptosis by EGCG is also exerted by the ER-independent actions with the inhibition of aryl hydrocarbon- (AhR-) regulated genes [103–106]. More recently, Baker and Bauer reported the antiproliferative mechanism of EGCG by blocking the ER*β*-specific inhibitor PHTPP [193]. In addition, EGCG is reported to induce the apoptosis in ER-negative MDA-MB-468 [104] and MDA-MB-231 cells [106, 107] and also alters the EGFR activity [108]. EGCG is also suggested to increase the protein expression of p21 and p27 [109], as well as enhanced the expression of proapoptotic genes, caspase-3, caspase-8, and caspase-9, and TP53 [110, 111]. Another important study demonstrated the inhibitory effects of EGCG on the arachidonic acid pathway by controlling COX-2 expression through minimizing the activity of COX-2 promoter via inhibition of nuclear factor kappaB (NF-kappaB) activation [112, 113]. However, studies also showed that EGCG has no effect on the aromatase activity [194]. Moreover it is suggested that, from clinical trial based studies of breast cancer, EGCG increases the sensitivity of ionizing radiation [114] and also shows protection against the toxic adverse effects of chemotherapy and radiotherapy [195].

Furthermore, in combination therapy, the presence of EGCG significantly increased the bioavailability of tamoxifen [115], 5-fluorouracil [116], and doxorubicin [117], and also single oral doses of ECCG up to 1600 mg were safe and very well tolerated [196]. For more detailed information regarding the effect of EGCG as adjuvant in cancer therapy, the readers are suggested to refer to Stearns et al. [117]. However, still various reports showed the undesirable interactions of EGCG with some anticancer drugs and therefore much clinical based research need to establish the effective role of EGCG as an adjuvant in breast cancer therapy.

### 5.5. Genistein

Soy isoflavones have been identified and proved as dietary components having a very potential and significant role in decreasing the incidence of various cancers [197]. Genistein, the predominant isoflavone content in soy products, has been known as cancer chemopreventive agent for various cancers [118, 198]. Genistein and other soy isoflavones have been found to be successful in controlling COX-2 expression and also to antagonizing AA for controlling PGE2 [118]. Genistein prevented inflammatory responses by inhibiting sPLA2 activity [119]. Soy isoflavones, especially genistein, minimizes COX-2 expression in MCF-7 breast cancer cells, which could be the mechanism underlying prevention of breast carcinogenesis [120]. Chung et al. [121] demonstrated that genistein inhibited TPA-induced COX-2 expression and transcriptional activity of NF-*κ*B in MCF10A human breast epithelial cells by blocking ERK mediated phosphorylation of p65. This study supported the chemopreventive effect of genistein against breast cancer. Due to having structural resemblance with the estradiol (E2), genistein binds and activates both ER*α* and ER*β* [122]. As a result, it is assumed that the estrogenic effect of soy isoflavones like genistein may potentially increase the risk of breast cancer in Western countries [199] and reduce the risk of breast cancer prevalence in Asian countries, as they consume high soy foods [200]. Similar studies done by Zhang and colleagues reported the lower breast cancer rate in North American women; those are consume soy products [201]. These studies thence concluded that genistein acts as ER modulator [202] like tamoxifen and raloxifene, which are the most used drugs in breast cancer treatment and prevention. It is also reported that genistein induced apoptosis via the upregulation of Bax and p21WAF1 proteins in MDA-MB-231 cell lines [123] and also downregulated the expression of caspase-3 [124].

Some other studies also provided the information about genistein inducing apoptosis. Shim et al. reported that genistein was capable of inducing apoptosis in MCF-7 cells by controlling calpain-caspase-7 and protein kinase activation cascade and apoptosis signaling kinase 1-p38 mitogen-activated protein kinase activation cascades, which involved a mechanism of release of Ca from the endoplasmic reticulum [125]. Sergeev also suggested that genistein targets Ca2+-dependent proteases in breast cancer cells, and the apoptotic mechanism followed by genistein is based on the cellular Ca2+ regulatory activity [203].

Genistein can also exert its anti-breast-cancer activity by inhibiting cell proliferation. Chen et al. reported that by inactivating the IGF-1R-PI3K/Akt pathway and reducing the Bcl-2/Bax mRNA and protein expressions genistein can block the cell proliferation [126].

Another study suggested that genistein can enhance G2/M arrest by activating the ATM/Chk2/Cdc25C/Cdc2 checkpoint pathway and eventually increases the radiosensitivity of both [127] ER+ and ER− breast cancer cells by an apoptosis pathway mediated by mitochondria.

Genistein can be effective to control early breast tumorigenesis by epigenetic regulation. It can regulate* p21* and* p16* by playing a role in histone modifications [128]. Studies also suggested that it can epigenetically restore* ERα* expression, which ultimately induces sensitivity of TAM-dependent antiestrogen therapeutic [204]. Another study reported that the anticancer effect of genistein on breast carcinoma can be because of its ability of demethylation and reactivation of methylation-silenced TSGs by interacting with the DNMT1 catalytic domain and blocking the expression of DNMT1 [129].

Vissac-Sabatier et al. 2003 [205] found that genistein can uprise the regulation of Brca1 and Brca2 mRNA expressions in adult ovariectomised rats. However, the outcome in mice inoculated with mammary tumour cells from conditional* Brca1*−/− mice showed that genistein is able to reduce the size of the tumours by 50%. This indicates that genistein can be protective in the absence of functional* Brca1* [130].

Several studies reported that genistein processes low oral bioavailability [206, 207], despite much attempts recently made to increase the oral bioavailability [208–210]; more clinical based research will be required before recommending the intake of genistein in breast cancer therapy.

### 5.6. Lycopene

Lycopene is the bright red carotene pigment belonging to tetra terpenoids and a phytochemical, which occurs naturally in tomato, carrot, watermelon, papaya, and cherry. Being a potent antioxidant it regulates multiple genes which are involved in DNA repair mechanism, control of cell cycle, and apoptosis in breast cancer cells [211, 212]. Studies by King-Batoon et al. [131] demonstrated the activity of lycopene on GSTP1 gene in breast cancer cells. It was observed that lycopene (2 *μ*M for 1 week) upregulated the expression of GSTP1 and has the ability to demethylate GSTP1 promoter in MDA-MB-468 cell line; however, the scenario is not similar in MCF-7 breast cancer cells. The expressions of other genes such as RAR*β*2 and HIN1 remained unaltered by lycopene treatment in MCF-7 and MDA-MB-468 breast cancer cells [131, 213]. In addition, lycopene can induce cell apoptosis and exert antitumor effects by regulating cell growth factor signaling pathways and thereby activate cell cycle arrest. Similar study done by Takeshima et al. [132] showed that lycopene potentially inhibited cell proliferation by blocking the phosphorylation of Akt following its downstream pathway and also upregulated the proapoptotic Bax without affecting antiapoptotic Bcl-xL in triple-negative breast cancer cells. Lycopene was also found to suppress cyclin D1 with the upregulation of p21 and also sustained the activation of the ERK1/2. After that, Peng et al. uncovered the antiproliferative mechanism of lycopene in MCF-7 cell lines, in which lycopene regulated the expression of p53 and Bax and thereby reduced the cell proliferation and increased apoptosis [133]. The mechanisms of lycopene in case of ER subtypes still remain controversial, where one group reported the negative correlation of lycopene and other carotenoids with ER (−) subtypes [214], while other groups demonstrated that consumption of carotenoids like lycopene could diminish the risk of the ER (−) subtypes [215]. Therefore, more studies are still necessary to elucidate chemopreventive mechanism of lycopene in other pathways as well as in different breast cancer subtypes. Besides, Rao and Shen [216] recommended a dose of lycopene with a range of 5 to 10 mg/day, which is effective to protect cell damaging from free radicals. Though in a clinical based study it is reported that supplementation nutritional extract containing lycopene reduced the oxidative stress and carcinogenesis in prostate cancer patients [217], still there is limited study of health benefits of lycopene alone, and further clinical trial based studies on the chemopreventive effect of lycopene are to be investigated.

### 5.7. Shikonin

Shikonin, mainly isolated from the root extract of* Lithospermum erythrorhizon, *has been reported to have anticancer, anti-inflammatory, wound healing, antiviral, and a wide range of biological effects [218]. In breast cancer pathway, shikonin was found to inhibit estrogen stimulated cell growth and initiates ER ubiquitination which in turn activates ER degradation in ER-positive breast cells [143]. Reports from the experiment conducted by Yao group demonstrated that shikonin inhibited pS2 and c-myc and estrogen responsive gene promoters in breast cancer cell and also make protection against estrogen induced DNA damage by triggering the Nrf2 pathway [144]. In ER-positive breast cancer cell, shikonin induces apoptosis with the characteristics of necroptosis [219] and also decreases the expressions of steroid sulfatase genes [145]. Multiple pathways like activation of caspase-3, suppression of the NF-*κ*B pathway, and apoptosis-related genes Bcl-2 and Bax modification are targeted by shikonin during the induction of apoptosis. Shikonin also suppresses NF-*κ*B pathway through the downregulation of p65 and inhibition of I*κ*B-*α* phosphorylation [146]. Jang et al. reported that shikonin blocked migration and invasion mechanism in breast cancer cell through modulating matrix metalloproteinase-9 (MMP-9) [220], while Wang et al. reported that shikonin suppress ER-negative human breast cancer cell growth by inhibiting the expression of HIF-1a [221]. Furthermore, shikonin also increases the chemosensitivity of taxol in ER-negative human breast cells, inducing the cell cycle arrest at the G2/M phase, and also inhibits the activation of ERK, Akt, and p70S6 kinases, which are the major player of cancer drug resistance [147].

Zhang et al. showed that shikonin decreases tamoxifen resistance by inducing uc.57 in MCF-7R breast cancer cells that inhibits PI3K/Akt and MAPK signaling pathways through downregulating BCL11A [148].* In vivo* pharmacokinetics studies represented shikonin as lower toxic [222], and this compound has potential to be considered further for the drug studies against breast cancer. However, shikonin results in poor bioavailability profiles and undergoes extensive first-pass metabolism [223]; therefore, extended studies are necessary to enhance the bioavailability profile of shikonin. And also, clinical application of shikonin requires more preclinical data, and clinical trials based study should be introduced to understand the efficacy of shikonin in single and adjuvant therapy in breast cancer.

### 5.8. Sulforaphane

Sulforaphane (SFN) is an isothiocyanate which is isolated from broccoli, water crass, broccoli sprouts, cabbage, and kale [224] and was found to inhibit proliferation, angiogenesis, and metastasis. At the same time it can induce cell cycle arrest and apoptosis in breast cancer cells. Studies by Bishayee [154] demonstrated that, in MCF-7 and MDA-MB-231 breast cancer cells, SFN treatment can result in the inhibition of hTERT (human telomerase reverse transcriptase) in both dose-dependent and time-dependent manner through an epigenetic pathway which include DNA methylation and histone modifications. Studies showed that SFN exerts chemopreventive action by inducing cell cycle arrest at G2/M phase through increasing the expression cyclin B1 and activates the poly(ADP-ribose) polymerase 1 and caspase family proteins followed by apoptosis in human breast cancer cell lines [134, 135]. SFN was also demonstrated to inhibit tubulin polymerization in breast cancer cells [136]. Recently, kim et al. suggested that treatment of SFN in breast cancer cell lines resulted in the downregulation of the nuclear factor kappa B signaling pathway. SFN also decreased the expressions of Bcl-2 and phosphorylated Akt serine/threonine kinase. This study also suggested that, in a combination therapy, SFN increases chemosensitivity of paclitaxel in breast cancer cells [137, 138]. SFN also causes the epigenetic modification by altering gene expressions of* hTERT* and estrogen receptor-*α (ERα)* [139, 190, 225]. Supporting this, Li et al. [139] demonstrated that SFN enhanced the sensitivity of tamoxifen by epigenetic reactivation of* ERα* in ER*α*-negative breast cancer, where the study was done both* in vitro* and* in vivo*. Another study demonstrated that SFN inhibits the expression of nuclear factor kappa B and COX-2 through blocking of signaling pathways mediated by ERK1/2-IKK-*α* and NAK-IKK- *β* [140]. It appeared from the study by Li et al. [142] that treatment of SFN in human breast cancer cell lines decreased the number and size of mammospheres as well as ALDH+ cell population, while, in NOD/SCID xenograft model, daily treatment of sulforaphane at dose of 50 mg/kg for 2 weeks decreased ALDH+ and downregulated the Wnt/*β*-catenin self-renewal pathways. Another study by Li group showed that SFN decreased the expressions of SOX9 and ALDH1 in a model of ER*α*-negative/basal-like DCIS and thereby eliminated tumor* in vivo* [141]. This group also observed that SFN caused the significant changes of DCIS stem-like cells in exosomal secretion which more closely resembles that of non-stem cancer cells [226]. These studies indicate that SFN could reprogram and eliminate CSCs [227]. In trail based studies, SFN was proved to produce chemopreventive effect in human breast tissue in a dose-dependent manner [178]. Furthermore, at low dietary dose, SFN shows high absolute bioavailability; however, increasing dose reduced the bioavailability [228], while SFN is well tolerated and causes no significant toxicity in humans and can reach effective concentrations in human plasma and tissue. Therefore, sulforaphane could be a very promising adjunct to chemotherapeutic drugs, especially given the fact that most of these drugs do not have the capability to eliminate CSCs and are often followed by tumor resistance and recurrence [229]. However, additional studies including larger population-based studies are needed to ensure the treatment and effect of SFN on chemopreventive modulation.

### 5.9. Resveratrol

Resveratrol (trans-3,4′,5-trihydroxystilbene), a plant derived polyphenolic compound, is present in grapes, which has been identified as potent antiaging, anti-inflammatory, and chemopreventive agent that affects various molecular targets [230, 231]. Qin et al. reported that resveratrol acts as DNMT 3b inhibitor and decreases in RASSF-1*α* methylation with increasing circulating resveratrol and it also suppresses expression of the androgen receptor [155]. By considering cellular targets related to epigenetic pathways, SIRT1 and acetyl transferase p300 were reported to be activated by resveratrol [154, 156]. In case of triple-negative breast cancer cells, combination of resveratrol and pterostilbene applied in a dose very close to physiologically relevant dose showed synergistic (CI < 1) growth inhibition by downregulating SIRT1. Further study done by Stefanska et al. [157] showed that resveratrol decreased the expressions of DNMT1, DNMT3a, and DNMT3b, HDAC1, and methyl CpG binding protein 2 (MeCP2) in MCF-7 cell line. Several studies also represented that resveratrol binds with some important molecular targets of breast cancer pathway, including estrogen receptor-*β* (ER*β*), cyclooxygenase-2 (COX-2), quinine reductase 2 (NQO2), inhibitor of kappa B kinase (IKK), and glutathione sulfotransferase (GSTP1), where COX-2 is involved in carcinogenesis/inflammation; ER*β* and IKK are involved in gene modulations and transcriptional regulation; NQO2 and GSTP1 are phase II detoxification enzymes [158]. It is thought that resveratrol exerts chemopreventive action by altering their stability, activity, and signal transduction [159]. Resveratrol also acts as a phytoestrogen and is observed to decrease the E2-stimulated cell growth and transcription process of PR [232], while Park et al. [233] observed that this antagonistic effect is only for ER*α* subtypes. A study reported by Bhat et al. showed that [234], in the presence of E2, resveratrol acts as antiestrogen and an agonist or antagonist in the absence of E2 in different breast cancer cell lines. On this side, a report from recent pilot clinical study, it has been observed that consumption of 1 g resveratrol per day had complimentary effects on estrogen metabolism as well as sex steroid hormone binding globulin in postmenopausal women having high body mass index [161]. In a dose-dependent manner, resveratrol was found to reduce aromatase mRNA and protein expression in SK-BR-3 cells and also suppressed the transactivation of CYP19 promoters I.3 and II [160]. Resveratrol also plays major roles in cellular apoptosis. Similarly, in MDA-MB 468 (basal subtype), resveratrol stimulates apoptosis through wild type p53-dependent pathway [162], while it promotes cell cycle arrest at S-phase and thereby induced apoptosis at a low dose in MCF-7 cells [163].

Pozo-Guisado et al. observed that resveratrol induced apoptosis by suppressing ER*α*- dependent PI3K pathway in MCF-7 cells [235], while in other cell lines, including MDA-MB-231, MDA-MB-453 (basal subtype), and MDA-MB-468, induction of apoptosis was caused by inhibiting Src tyrosine kinase and signal transducer and activator of transcription 3 (STAT-3) phosphorylation pathways [236]. Reduction of Akt phosphorylation and activation of procaspase-9 was also observed in MCF-7 cells caused by resveratrol to induce apoptosis [164].

By upregulating Ca+2 leakage and calpain activity in MCF-7 cells, resveratrol caused programmed cell death in such a mechanism that is not controlled by caspase, whereas, in MDA-MB-231 cells, resveratrol can ultimately increase mitochondrial membrane potential to breakdown and release of cytochrome* c* (cyt.* c*). Ultimately, mitochondria-derived activator of caspases or direct inhibitor of apoptosis (IAP) binding protein is activated with mitochondrial protein (Smac/DIABLO), caspase-9, and caspase-3 and thereby apoptosis is induced [165]. The cytotoxicity of tumor necrosis factor- (TNF-) related apoptosis-inducing ligand (TRAIL) can be induced by resveratrol and can also be used as an adjuvant for TRAIL-based therapies in order to minimize TRAIL that is required for suppressing growth of tumor. It also has the potential to sensitize resistant tumors to TRAIL induced apoptosis [237].

Though resveratrol illustrates potent anti-breast-cancer activity by regulating a number of significant pathways, it needs more attentions regarding the improvement of bioavailability, as it shows less oral absorption rate [238, 239]. Furthermore, the chemopreventive action of resveratrol still needs to be confirmed by future clinical trials; also a vast amount of clinical data is necessary to understand the therapeutic potential of resveratrol, and more study is needed to develop its derivatives that provide better bioavailability and is effective in different types of breast cancer patient [240, 241].

Apart from the above-mentioned natural compounds, there are some other chemical compounds currently being focused on and investigated for their promising chemopreventive potential in breast cancer. Similarly, silibinin is a flavonolignan, extracted from milk thistle (*Silybum marianum*), and has been reported to provoke autophagic cell death in breast cancer cells by downregulating the Bcl-2 expression and upregulation of Atg12-Atg5 formation and enhancing beclin-1 expression [149]. Karimi et al. [242] Represented that silibinin exhibits cytotoxic activity in breast cancer cell line through reducing the expression of ER*α*. The anticancer activity of silibinin is associated with the suppression Wnt/LRP6 signaling [150] and also downregulates the TPA-induced MMP-9 expression through inhibiting COX-2 expression in breast cancer cells [151]. In adjuvant therapy, silibinin is reported to increase the efficacy of cisplatin and paclitaxel in MCF-7 breast cancer cells [152] and also to sensitize the chemoresistant breast cancer cells [153]. Similarly, quercetin glycoside such as quercetin 3-O-*β*-d-rutinoside (rutin) is reported to promote apoptosis in breast cancer cell line, while restoring the chemosensitivity in HER2-negative and triple-negative breast cancer cell lines [168]. Elsayed et al. [169] reported that rutin inhibits c-Met kinase activity in TNBC cell line and also significantly reduces the growth of TNBC MDA-MB-231/GFP orthotopic xenograft in nude mouse model at a dose of 30 mg/kg. Emodin was reported to inhibit HER-2/neu tyrosine kinase activity in HER-2/neu-overexpressing cancer cells [99] and activates apoptosis through disruption of mitochondrial signaling pathway [243, 244]. In another study, it appeared that a natural phytocompound from rosemary, rosmarinic acid, inhibits breast cancer cell proliferation through reducing the COX-2 expression, AP-1 activation, and antagonized the ERK1/2 activation [166], while Wang et al. showed that rosmarinic acid suppresses interleukin-8 (IL-8) in the pathway of the NF-*κ*B and thereby inhibits bone metastasis from breast carcinoma [167].

According to these studies, these phytochemicals have been shown potent to ameliorate the anticancer therapy in breast cancer (Table 1). However, more extensive studies are needed to conduct and authenticate these promising anticancer and chemopreventive compounds and their activities in clinical stage.

## 6. Future Prospect of Herbal Management

Presently the traditional treatment approaches that are being used for breast cancer treatment are being interrupted by a number of impediments mainly for the toxic effects accompanied by drug resistance. Chemotherapy or radiotherapy is creating a number of adverse effects in patients which are inevitable. Again due to drug resistance, the responses of these therapies become poor. In this case natural compounds from dietary sources can be blessings, in view of the fact that they can show synergistic action with many chemotherapeutics and can induce the efficacy of them. A number of natural compounds are reported to have a very positive outcome if taken with other medicines in breast cancer treatment. Following this strategy, a number of medicines and dietary components combination are reported. Among them some examples are combination of genistein and doxorubicin provides a synergistic effect [245], equol induces the efficacy of tamoxifen, pomegranate also reported to increase cell death and enhance tamoxifen-induced inhibition on cell viability [246], and DIM works synergistically with Paclitaxel and helps to induce apoptosis [247]. Rosemary extract can increase activity of anti-breast-cancer agent tamoxifen, trastuzumab, and paclitaxel [248]. These can be great fields for future research on natural compounds and can be a successful alternative approach for treating breast cancer.

Chemotherapy is the most popular and most important treatment strategy of cancer; however, there is a rising problem that patient can be unable to respond to chemotherapy due to chemoresistance [249]. This resistance can be developed by the activity of ATP-binding cassette (ABC) transporters that pump anticancer drugs out of the cells [250]. Hence a number of researchers are trying to find out natural compounds which act against multidrug resistance. *β*-elemene is a natural compound acting on MDR in human breast carcinoma MCF-7 and doxorubicin-resistant MCF-7 cells and can be a very promising agent for facing the problem of multidrug resistance [251]. Another research work reported that 3,3′-diindolylmethane (DIM) can function as a radio sensitizer to multidrug resistance breast cancer cells and eventually can fight against multidrug resistance [173]. So more research works on nature derived chemicals are necessary to find out their pathway and their possible role on acting against MDR.

The most aggressive subtype of breast cancer is triple-negative breast cancer and its treatment options are very limited as there is lack of a therapeutic target. Therefore, scientists are now trying to find out novel targets as well as alternative treatments for TNBC. A number of natural compounds are investigated and some of them showed satisfactory results. Curcumin and resveratrol are studied [158, 252] and it is evident that both of these compounds act effectively to treat TNBC and can also reduce side effects [253]. A natural compound EGCG, derived from green tea, is reported to block uncontrolled cell growth and can inhibit the migratory behavior of triple-negative breast cancer [254]. In a recent work, carnosol, a naturally occurring compound is reported to block cell cycle at G2 phase and also found to increase ROS-dependent apoptosis and beclin-1-independent autophagy in triple-negative MDA-MB-231 human breast cancer cells [255]. This proves that carnosol can be used to design a medicine for TNBC. Natural compounds can thus be used as a treatment system to fight TNBC.

In this review, some natural compounds are highlighted which are potent to modify any mechanism related to breast cancer. Nevertheless, there are some compounds which can act in more than one mechanism. As a result, they can show greater degree of efficacy. Again some of these compounds can show synergistic activity or they can work against multidrug resistance. By considering all these factors, it can be said that natural compounds can play a very promising role in treatment and prevention of breast cancer in near future if more deep studies are conducted.

## 7. Conclusion

The significance and contribution of natural derived compounds in the treatment and prevention of breast cancer is evident and cannot be ignored. The applications of the nature derived dietary chemicals are mostly widespread, and they are being practiced traditionally from a long time. More emphasis must be given on research works to optimize the activity of these compounds and to develop them as a therapeutic to treat the patient with breast cancer. These natural compounds can potentiate the activity of other traditional treatments and can also be used as a treatment system alone as they have the ability to modify different mechanisms. Apart from this, they play a strong role in preventing breast cancer. They can function by a number of pathways without initiating any kind of unusual toxic effect. In this review, the epigenetic mechanism, aromatase activity, arachidonic acid pathway, and apoptosis pathway are discussed and it is also evident that they have relationship with development of breast cancer. A number of natural compounds are highlighted in this work and their mode of action, pathway, synergistic action, and their future potential are widely discussed. Therefore, more detailed research works are required to confirm the appropriate role of these chemicals which can give us better understanding on their therapeutic use and can help us fight the existing challenges of breast cancer.

## Figures and Tables

**Table 1:** Summary of activities of natural compounds in breast cancer treatment and management.

Chemical compound	Structure	Major source	Mechanisms	Remarks	References
3,3′-Diindolylmethane	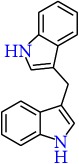	Broccoli, cauliflower, cabbage	Arachidonic acid pathway	(i) Inhibits COX-2 expression in MCF-7 breast cancer cells	[83]
			Apoptosis pathway	(i) Downregulates survivin, Bcl-2, and cdc25A (ii) Upregulates p21(WAF1) expression	[84]
			HIF-1 signaling pathway	(i) Decreases the expression of key hypoxia responsive factors, VEGF, furin, enolase-1, glucose transporter-1, and phosphofructokinase in hypoxic tumor cell lines	[85]
			Aromatase activity	(i) Inhibits aromatase expression	[86]
			Chemosensitivity/ adjuvant therapy	(i) Increases the efficacy of herceptin by reducing *FoxM1, Akt,* and *NF-κB p65 level* (ii) Increases the chemosensitivity of tamoxifen and alter the estrogen metabolism in randomized, placebo-controlled trial (iii) Sensitizes MDR human breast carcinoma to *γ*-irradiation	[87–89]

Biochanin A	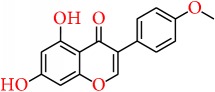	Red clover	Aromatase activity	(i) Inhibits aromatase expression	[90]
			Cytoplasmic signaling pathways	(i) Inhibits HER-2 receptor activation (ii) Inhibits Erk1/2, Akt, mTOR, *NF-κB*, MMP-9, and MT-MMP1	[91]
				(i) Inhibits tumor growth in a xenograft animal model at dose of 15 mg/kg	[92]

Curcumin	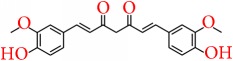	Turmeric	Apoptosis pathway	(i) Increases p53 level (ii) Increases Bax expression (iii) Downregulates NF-kappaB, cyclin D, and MMP-1 transcription in MDA-MB-231 and BT-483 cell line	[93]
			Wnt signaling pathway	(i) Inhibits Wnt signaling at a dose of 5 *μ*M in MCF-7 cell line	[94]
			Epigenetic regulation	(i) Inhibits the expression of class I HDACs (ii) Upregulates the expression of some miRNAs to reduce the expression of Bcl-2	[95, 96]
			Chemosensitivity/ adjuvant therapy	(i) Enhances the efficacy of paclitaxel by deactivating NF-*κ*B and MMP-9 expressions in MDA-MB-231 cell (ii) Inhibits the efflux function of P-gp, MXR, and MRP1 in MCF-7 and MCF-7MDR cell line	[97, 98]

Emodin	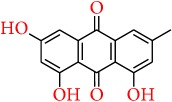	Rhubarb, buckthorn	Apoptosis pathway	(i) Inhibits HER-2/neu tyrosine kinase activity in HER-2/neu-overexpressing cancer cells	[99]

Epigallocatechin gallate	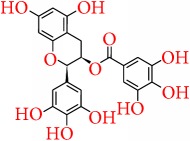	Green tea	Epigenetic regulation	(i) Decreases 5-methylcytosine, DNA methyltransferase (DNMT) activity, DNMT1, DNMT3a, and DNMT3b (ii) Decreases histone deacetylase activity (iii) Increases levels of acetylated lysine 9 and 14 on histone H3 (H3-Lys 9 and 14) and acetylated lysine 5, 12, and 16 on histone H4 (iv) Decreases the levels of methylated H3-Lys 9. (v) Increases the expression of p16INK4a and Cip1/p21. (vi) Induces the expression of epigenetically repressed TIMP-3 gene	[100–102]
			Apoptosis pathway	(i) Decreases aryl hydrocarbon- (AhR-) regulated genes (ii) Blocks ER*β*-specific inhibitor PHTPP (iii) Decreases the expression of Bcl-2 but increases Bax (iv) Increases release of cytochrome c (v) Increases the expression of Apaf-1 (vi) Activates of caspase-3 and poly(ADP-ribose) polymerase (vii) Alters the EGFR activity (viii) Increases the expression of p21 and p27, caspase-3, caspase-8, and caspase-9 and TP53	[103–111]
			Arachidonic acid pathway	(i) Decreases the COX-2 expression and kappaB (NF-kappaB) activations	[112, 113]
			Chemosensitivity/ adjuvant therapy	(i) Increases the sensitivity of ionizing radiation (ii) Increases the bioavailability of tamoxifen, 5-fluorouracil, and doxorubicin	[114–117]

Genistein	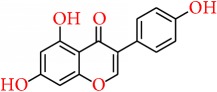	Soy products	Arachidonic acid pathway	(i) Inhibits COX-2 expression (ii) Regulates PGE2 (iii) Inhibits sPLA2, NF-*κ*B, and ERK mediated phosphorylation of p65 in breast cancer cell (iv) Activates both ER*α* and ER*β*	[118–122]
			Apoptosis pathway	(i) Upregulation of Bax and p21WAF1 protein in MDA-MB-231 cell lines (ii) Downregulation of caspase-3 (iii) Regulates calpain-caspase-7 and 1-p38 mitogen-activated protein kinase activation cascades (iv) Inactivates the IGF-1R-PI3K/Akt pathway and reducing the Bcl-2/Bax (v) Enhances G2/M arrest by activating the ATM/Chk2/Cdc25C/Cdc2 checkpoint pathway	[123–127]
			Epigenetic regulation	(i) Regulates the p21 and p16 expression (ii) Epigenetically restores ER*α* expression (iii) Blocks the expression of DNMT1	[128, 129].
				(i) Uprises the regulation of Brca1 and Brca2 mRNA expressions in adult ovariectomised rats (ii) Reduces the size of the tumours by 50%	[130]

Lycopene	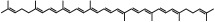	Tomato, carrot, watermelon, papaya, cherry	Epigenetic regulation	(i) Upregulated the expression of GSTP1 (ii) Demethylates GSTP1 promoter	[131]
			Apoptosis pathway	(i) Blocks the phosphorylation of Akt downstream pathway (ii) Upregulates the proapoptotic Bax without affecting antiapoptotic Bcl-xL (iii) Suppresses cyclin D1 and upregulates p21 (iv) Sustains the activation of the ERK1/2 (v) Increases the expression of p53 and Bax	[132, 133].

Sulforaphane	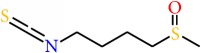	Broccoli, water crass, boccoli sprouts, cabbage, kale	Epigenetic regulation	(i) Inhibits hTERT (human telomerase reverse transcriptase)	[134]
			Apoptosis pathway	(i) Increases the expression cyclin B1 (ii) Activates the poly(ADP-ribose) polymerase 1 and caspase family proteins (iii) Inhibits tubulin polymerization (iv) Downregulates the nuclear factor kappa B signaling pathway (v) Decreases the expressions of Bcl-2 and phosphorylated Akt serine/threonine kinase	[134–136]
			Chemosensitivity/ adjuvant therapy	(i) Increases chemosensitivity of paclitaxel in breast cancer cells (ii) Enhances the sensitivity of tamoxifen by epigenetic reactivation of ER*α* in ER*α*-negative breast cancer	[137–139]
			Arachidonic acid pathway	(i) Inhibits the expression of nuclear factor kappa B (ii) Blocks COX-2 expression, which is mediated by ERK1/2-IKK-*α* and NAK-IKK- *β*	[140, 141]
				(i) Decreases the expressions of SOX9 and ALDH1 in vivo (ii) Decreases ALDH+ and downregulates the Wnt/*β*-catenin self-renewal pathways in NOD/SCID xenograft model at a dose of 50 mg/kg for 2 weeks	[141, 142]

Shikonin	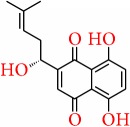	*Lithospermum erythrorhizon*	Estrogen signaling	(i) Activates ER ubiquitination (ii) Inhibits pS2 and c-myc, estrogen responsive gene promoters (iii) Induces DNA damage by triggering the Nrf2 pathway	[143, 144]
			Apoptosis pathway	(i) Decreases the expressions of steroid sulfatase genes (ii) Activates caspase-3 (iii) Suppresses NF-*κ*B pathway, Bcl-2, and Bax (iv) Downregulates p65 and inhibition of I*κ*B-*α* phosphorylation	[145, 146]
			Chemosensitivity/ adjuvant therapy	(i) Increases the chemosensitivity of taxol in ER negative human breast cells (ii) Induces cell cycle arrest at the G2/M phase and inhibits the activation of ERK, Akt, and p70S6 kinases (iii) Decreases tamoxifen resistance by inducing uc.57 and downregulates BCL11A	[147, 148]

Silibinin	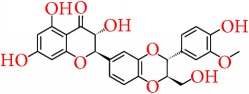	Milk thistle	Apoptosis pathway	(i) Induces autophagic cell death by downregulating the Bcl-2 expression (ii) Upregulates Atg12-Atg5 formation and enhances beclin-1 expression	[149]
			Arachidonic acid pathway	(i) Suppresses Wnt/LRP6 signaling (ii) Downregulates the TPA-induced MMP-9 expression and inhibits COX-2 expression in breast cancer cells	[150, 151]
			Chemosensitivity/ adjuvant therapy	(i) Increases the efficacy of cisplatin and paclitaxel	[152, 153].

Resveratrol	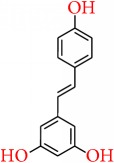	Grapes	Epigenetic regulation	(i) Inhibits DNMT 3b expression and decreases RASSF-1*α* methylation (ii) Activates SIRT1 and acetyl transferase p300 (iii) Decreases the expressions of DNMT1, DNMT3a, and DNMT3b, HDAC1, and methyl CpG binding protein 2 (MeCP2) in MCF-7 cell line	[154–157]
			Arachidonic acid pathway	(i) Inhibits ER*β*, COX-2, NQO2, IKK, and GSTP1	[158, 159]
			Aromatase activity	(i) Reduces aromatase mRNA expression (ii) Suppresses the transactivation of CYP19 promoters I.3 and II	[160]
				(i) Consumption of 1 g resveratrol per day had complimentary effects on estrogen metabolism as well as sex steroid hormone binding globulin in postmenopausal women having high body mass index	[161]
			Apoptosis pathway	(i) Stimulates p53-dependent pathway at a low dose in MCF-7 cells (ii) Suppresses ER- dependent PI3K pathway (iii) Src tyrosine kinase and signal transducer and activator of transcription 3 (STAT-3) phosphorylation pathways (iv) Reduces Akt phosphorylation and activates of procaspase-9 (v) Activates mitochondrial protein (Smac/DIABLO), caspase-9, and caspase-3	[162–165]

Rosmarinic acid	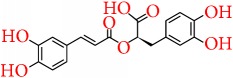	Rosemary	Arachidonic acid pathway	(i) Reduces the COX-2 expression, AP-1 activation, and antagonized the ERK1/2 activation (ii) Suppresses interleukin-8 (IL-8) in the pathway of the NF-*κ*B	[166, 167]

Rutin (quercetin 3-O-*β*-d-rutinoside)	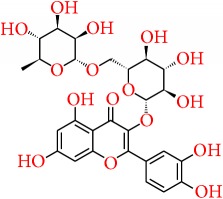	Buckwheat	Chemosensitivity/ adjuvant therapy	(i) Restores the chemosensitivity in HER2-negative and triple-negative breast cancer cell lines (ii) Reduces c-Met kinase activity in TNBC cell line and also significantly reduces the growth of TNBC MDA-MB-231/GFP orthotopic xenograft in nude mouse model at a dose of 30 mg/kg	[168, 169]
